# Near infrared photoimmunotherapy targeting bladder cancer with a canine anti-epidermal growth factor receptor (EGFR) antibody

**DOI:** 10.18632/oncotarget.24876

**Published:** 2018-04-10

**Authors:** Tadanobu Nagaya, Shuhei Okuyama, Fusa Ogata, Yasuhiro Maruoka, Deborah W. Knapp, Sophia N. Karagiannis, Judit Fazekas-Singer, Peter L. Choyke, Amy K. LeBlanc, Erika Jensen-Jarolim, Hisataka Kobayashi

**Affiliations:** ^1^ Molecular Imaging Program, Center for Cancer Research, National Cancer Institute, National Institutes of Health, Bethesda, Maryland, USA; ^2^ Purdue University Center for Cancer Research, Purdue University, West Lafayette, Indiana, USA; ^3^ St. John’s Institute of Dermatology, School of Basic and Medical Biosciences, King’s College London, London, UK; ^4^ Breast Cancer Now Research Unit, School of Cancer and Pharmaceutical Sciences, King’s College London, Guy’s Cancer Centre, London, UK; ^5^ Comparative Medicine, The Interuniversity Messerli Research Institute, University of Veterinary Medicine Vienna, Medical University Vienna and University Vienna, Vienna, Austria; ^6^ Institute of Pathophysiology and Allergy Research, Center of Pathophysiology, Infectiology and Immunology, Medical University Vienna, Vienna, Austria; ^7^ Comparative Oncology Program, National Cancer Institute, National Institutes of Health, Bethesda, Maryland, USA

**Keywords:** near infrared photoimmunotherapy, bladder cancer, canine cancer, EGFR, TCC

## Abstract

Anti-epidermal growth factor receptor (EGFR) antibody therapy is used in EGFR expressing cancers including lung, colon, head and neck, and bladder cancers, however results have been modest. Near infrared photoimmunotherapy (NIR-PIT) is a highly selective tumor treatment that employs an antibody-photo-absorber conjugate which is activated by NIR light. NIR-PIT is in clinical trials in patients with recurrent head and neck cancers using cetuximab-IR700 as the conjugate. However, its use has otherwise been restricted to mouse models. This is an effort to explore larger animal models with NIR-PIT. We describe the use of a recombinant canine anti-EGFR monoclonal antibody (mAb), can225IgG, conjugated to the photo-absorber, IR700DX, in three EGFR expressing canine transitional cell carcinoma (TCC) cell lines as a prelude to possible canine clinical studies. Can225-IR700 conjugate showed specific binding and cell-specific killing after NIR-PIT on EGFR expressing cells *in vitro*. In the *in vivo* study, can225-IR700 conjugate demonstrated accumulation of the fluorescent conjugate with high tumor-to-background ratio. Tumor-bearing mice were separated into 4 groups: (1) no treatment; (2) 100 µg of can225-IR700 i.v. only; (3) NIR light exposure only; (4) 100 µg of can225-IR700 i.v., NIR light exposure. Tumor growth was significantly inhibited by NIR-PIT treatment compared with the other groups (*p* < 0.001), and significantly prolonged survival was achieved (*p* < 0.001 vs. other groups) in the treatment groups. In conclusion, NIR-PIT with can225-IR700 is a promising treatment for canine EGFR-expressing cancers, including invasive transitional cell carcinoma in pet dogs, that could provide a pathway to translation to humans.

## INTRODUCTION

Near infrared photoimmunotherapy (NIR-PIT) is a newly developed cancer treatment that employs a highly targeted mAb-photo-absorber conjugate (APC). The photo-absorber, IRDye700DX (IR700, silica-phthalocyanine dye), is a highly hydrophilic dye, differentiating it from prior hydrophobic dyes used in photodynamic therapy (PDT) [1]. A first-in-human trial of epidermal growth factor receptor (EGFR) targeted NIR-PIT in patients with inoperable head and neck cancer was initiated in June 2015 (https://clinicaltrials.gov/ct2/show/NCT02422979) and has recently completed Phase 2 testing. In this trial, patients were injected with cextuximab-IR700 conjugate, (referred to as RM1929 in the study), that binds EGFR on the cell membrane of head and neck cancers. About 24 hours later the tumor is exposed to NIR light by means of a laser at a wavelength of 690 nm which is absorbed by IR700. NIR-PIT using cetuximab-IR700 conjugate (RM1929) could be applied to other EGFR-expressing cancers in the body such as lung, colon, breast, esophagus, and bladder cancers.

In the United States (US), bladder cancer is the fourth most common cancer, with 79,000 estimated new cases and 17,000 bladder cancer related deaths in 2017 [2] and related to renally secreted toxins for instance from cigarette smoking [3]. At the time of diagnosis, most patients present with non-invasive bladder cancer, which has a 50% to 70% rate of superficial recurrence and a 10% to 30% rate of progression to invasive bladder cancer [4, 5]. Invasive bladder cancer outcomes remain poor despite aggressive multimodal treatment, with less than 10% survival at 5 years [6]. Further, bladder cancer is a disease of the elderly with coexistent medical conditions, and consequently approximately 50% of patients will be ineligible for cisplatin-based chemotherapy [7]. New highly targeted cancer therapies with improved side effect profiles for targeting invasive bladder cancer are urgently needed.

Studies in relevant animal models are essential to improve the management of invasive bladder cancer in human. Several experimentally induced models of transitional cell carcinoma (TCC; also referred to as invasive urothelial carcinoma) have been established, including chemically induced tumors, transgenic mouse models, and orthotopic xenograft models [8–10]. Although these experimental animal models are of great value and are constantly in use for invasive bladder cancer research, there is a need for complementary larger animal models in which the disease is naturally occurring, invades and metastasizes consistently, and more closely mimics the human condition. This will permit the development of instrumentation suitable for translation to humans and will improve the understanding of treatment effects. Dogs with naturally occurring invasive TCC provide an ideal animal model to address this need [11–13].

There are approximately 70 million pet dogs in the US, and it is estimated that 20 to 30,000 of these dogs develop bladder cancer each year [14]. In household dogs, topical insecticides and obesity seem to be more important than second hand smoke [15]. Naturally occurring bladder cancer in dogs very closely mimics human invasive bladder cancer, specifically high-grade invasive TCC in regard to histological appearance, cellular and molecular features, biological behavior including metastasis, and response to chemotherapy [11, 14]. In addition, molecular markers that have been implicated to be of importance in human invasive TCC, have also played a similar role in dogs [12, 13]. Thus, pet dogs offer a unique naturally occurring model of invasive bladder cancer, where response to new cancer therapies can be assessed and promising results translated into humans. In addition, the compressed life span of dogs makes the study of bladder cancer feasible from its earliest stages to death of the host in a timely manner, and the intact host immunity of the tumor-bearing pet dog allows assessment of immunomodulatory therapies from both efficacy and toxicity standpoints.

NIR-PIT has been shown to be effective with a variety of different antibodies, but has not been previously tested with a canine antibody and canine cancer models [16–21]. The purpose of this work was to establish NIR-PIT for canine invasive TCC *in vitro* and *in vivo* using the canine antibody can225IgG, which was constructed based on the exact EGFR-specific antigen-binding site of cetuximab [22] for comparative trials [23], and showed comparable affinity to the canine EGFR, which is 95% homologous to human EGFR [24]. Using three EGFR-expressing invasive canine TCC cell lines, K9TCC original (TCC original; minimal EGFR expression), K9TCC-PU AxA (TCC AXA; low EGFR expression) and K9TCC-PU Sh (TCC SH; intermediate EGFR expression), *in vitro* binding and *in vitro* NIR-PIT effects were evaluated. After *in vivo* tumor accumulation and intratumoral distribution were evaluated, NIR-PIT was then performed in a tumor-bearing mouse model *in vivo*. These experiments serve as a basis and provide rationale for moving forward into an actual clinical trial in privately-owned pet dogs with spontaneous cancers.

## RESULTS

### *In vitro* characterization of three TCC cell lines

**Figure 1:**
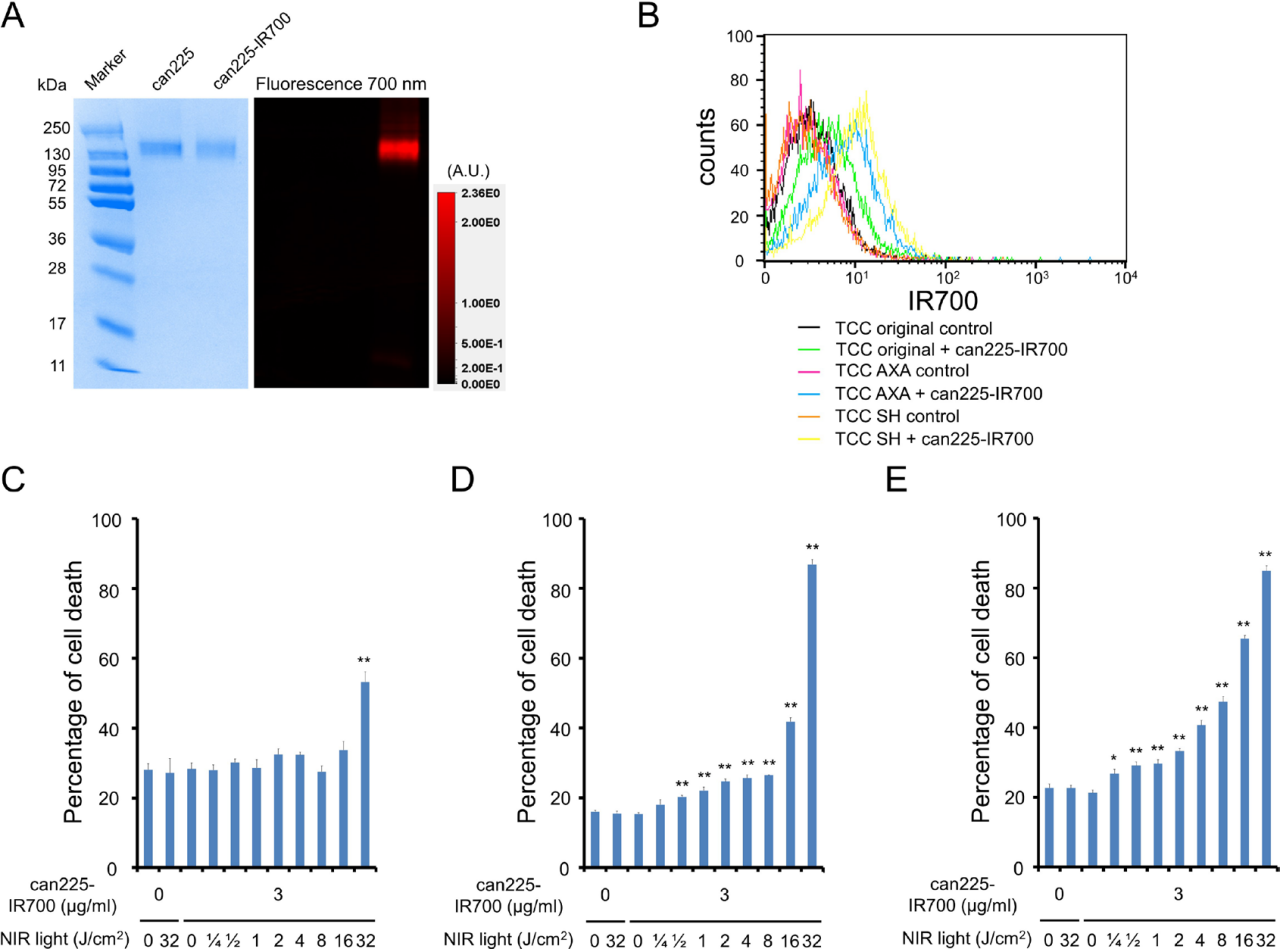
Confirmation of EGFR expression as a target for NIR-PIT in three TCC cell lines, and evaluation of *in vitro* NIR-PIT (**A**) Validation of can225-IR700 by SDS-PAGE (left: Colloidal Blue staining, right: fluorescence). Diluted can225IgG was used as a control. (**B**) Expressions of EGFR in three TCC cell lines were evaluated by flow cytometry. After 6 h of can225-IR700 incubation, TCC SH cells showed high fluorescence signal, TCC AXA cells showed intermediate fluorescence signal, and TCC original cells showed low fluorescence signal. Fluorescence in three TCC cell lines was completely blocked by adding excess can225IgG. (**C**) Membrane damage of TCC original cells induced by NIR light exposure was measured with the dead cell count using propidium iodide (PI) staining. No membrane damage was observed in TCC original cells after 0.25–16 J/cm^2^ of NIR light exposure. Membrane damage was only shown after 32 J/cm^2^ of NIR light exposure (*n* = 5, ^**^p < 0.01, vs. untreated control, by Student’s *t* test). (**D**) Membrane damage of TCC AXA cells induced by NIR-PIT was measured with the dead cell count using PI staining, which increased in a light dose dependent manner (*n* = 5, ^**^p < 0.01, vs. untreated control, by Student’s *t* test). There was no significant cytotoxicity associated with NIR light exposure alone in the absence of can225-IR700 and with can225-IR700 alone without NIR light exposure. (**E**) Membrane damage of TCC SH cells induced by NIR-PIT was measured with the dead cell count using PI staining, which increased in a light dose dependent manner (*n* = 5, ^**^p < 0.01, vs. untreated control, by Student’s *t* test). The NIR-PIT effects in TCC SH cells were higher than TCC original and TCC AXA cells. There was no significant cytotoxicity associated with NIR light exposure alone in the absence of can225-IR700 and with can225-IR700 alone without NIR light exposure.

As defined by sodium dodecyl sulfate-polyacrylamide gel electrophoresis (SDS-PAGE), can225-IR700 and non-conjugated control can225IgG showed an identical molecular weight, around 150 kDa, and fluorescence intensity was confirmed in the band of can225-IR700 (Figure 1A). After a 6 h incubation with can225-IR700, TCC SH cells demonstrated high fluorescence signal, which was confirmed with flow cytometry (Figure 1B). TCC AXA cells also showed intermediate fluorescence signal (Figure 1B). On the other hand, TCC original cells showed only slight fluorescence signal. Fluorescence signal in all three TCC cell lines was completely blocked by adding excess can225IgG (Figure 1B), confirming that can225-IR700 specifically binds to EGFR on the cells.

### *In vitro* NIR-PIT

Based on Propidium iodide (PI) incorporation, minimal NIR-PIT effects were observed in TCC original cells when 32 J/cm^2^ of NIR light was applied (Figure 1C). On the other hand, percentage of cell death increased in a light dose dependent manner in both TCC AXA (Figure 1D) and TCC SH cells (Figure 1E). Over 60% of TCC SH cells died when exposed to 16 J/cm^2^ of NIR light. There was no significant cytotoxicity associated with NIR light exposure alone in the absence of can225-IR700 or with can225-IR700 alone without NIR light exposure.

**Figure 2:**
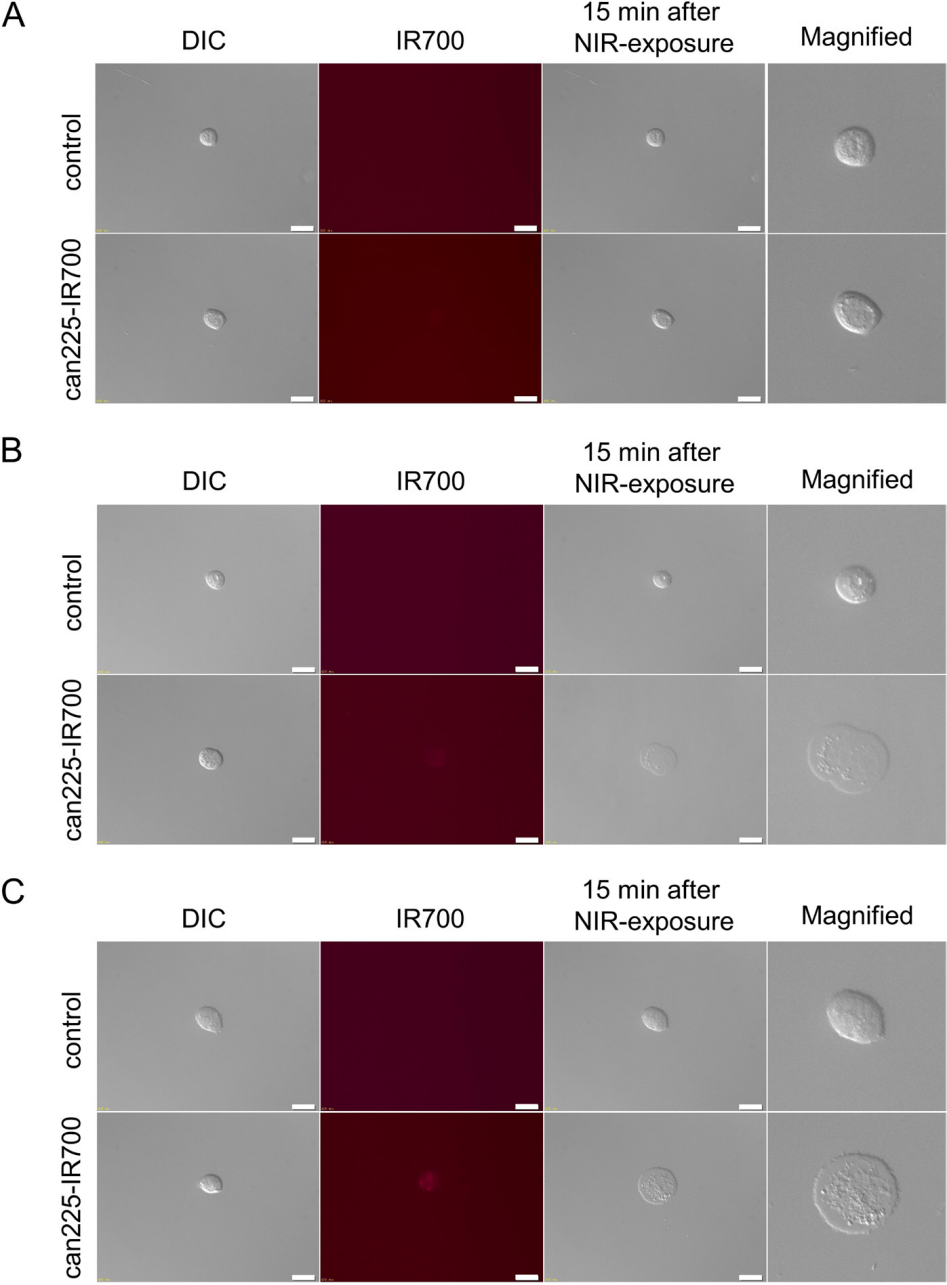
*In vitro* fluorescence microscopic images of three TCC cell lines (**A**) Differential interference contrast (DIC) and fluorescence images of TCC original cells after incubation with can225-IR700 for 6 h. Slight fluorescence signal was shown in TCC original cell. There was no cytotoxicity upon excitation with low dose NIR light (after 15 min) in TCC original cell. White scale bars = 20 µm. (**B**) DIC and fluorescence images of TCC AXA cells after incubation with can225-IR700 for 6 h. Intermediate fluorescence signal was shown in TCC AXA cell. Necrotic cell death was observed upon excitation with low dose NIR light (after 15 min) in TCC AXA cell. (**C**) DIC and fluorescence images of TCC SH cells after incubation with can225-IR700 for 6 h. High fluorescence signal was shown in TCC SH cell. Necrotic cell death was observed upon excitation with low dose of NIR light (after 15 min) in TCC SH cell.

In fluorescence microscopic studies, slight fluorescence signal was shown in TCC original cell, however, there was no cytotoxicity upon excitation with NIR light in TCC original cell (Figure 2A). On the other hand, NIR light exposure induced cellular swelling, bleb formation, and rupture of vesicles consistent with necrotic cell death in TCC AXA and TCC SH cells. Most of these morphologic changes were observed within 15 min of NIR light exposure (Figure 2B and 2C), indicating rapid induction of necrotic cell death. From these *in vitro* results, we selected TCC SH cells for *in vivo* study.

### *In vivo* fluorescence imaging studies

**Figure 3:**
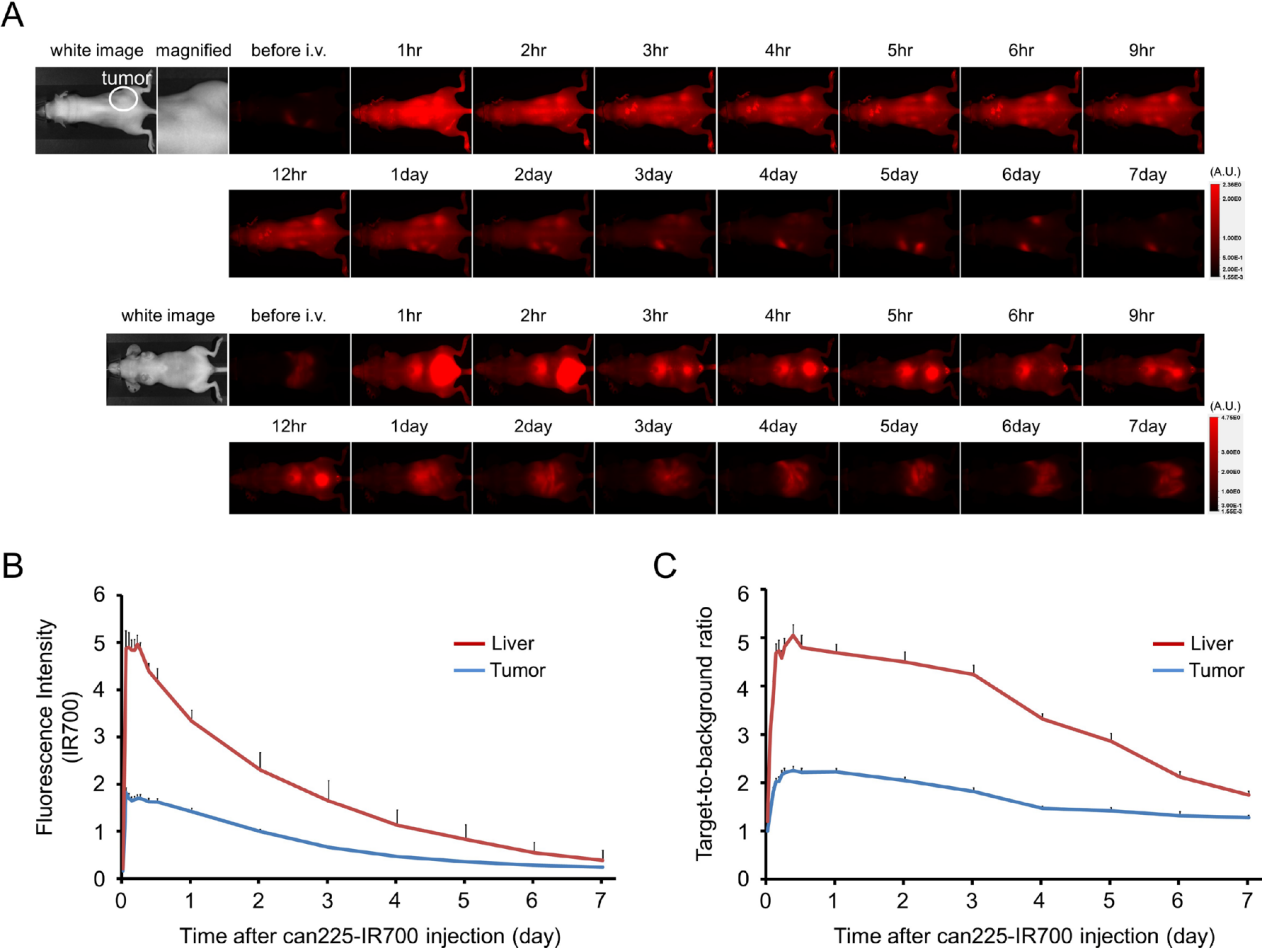
*In vivo* fluorescence imaging of TCC SH tumor (**A**) *In vivo* can225-IR700 fluorescence real-time imaging of tumor-bearing mice of TCC SH (right dorsum). The tumor showed high fluorescence intensity after injection and the intensity was gradually decreased over days. Most of the excess agent was excreted to the urine immediately after injection. (**B**) Time course of NIR fluorescence signal of IR700 in tumors and livers (*n* = 10). The IR700 fluorescence intensity of tumor showed high intensities within 1 day after can225-IR700 injection but this decreased gradually over days. The IR700 fluorescence intensity of liver showed high within 9 hours after APC injection, then this decreased. (**C**) Time course of NIR fluorescence signal of target-to-background ratio (TBR) in tumors and livers (*n* = 10). TBR of both tumor and liver showed high within three days after can225-IR700 injection, then the TBR was gradually decreased over the following days.

The fluorescence intensity of can225-IR700 in TCC SH tumor was high within 1 day after can225-IR700 injection but decreased over the following days (Figure 3A and 3B). The fluorescence signal in the liver was high for the first 9 hours after APC injection but then decreased (Figure 3A and 3B). On the other hand, the TBR of can225-IR700 in tumor and liver was high within 1 day after APC injection, following which the TBR did not change clearly for several days, then decreased gradually (Figure 3C). TBR of can225-IR700 was high in tumor due to specific APC binding to EGFR expressing TCC SH cells, while TBR was high in liver likely due to non-specific accumulation of can225-IR700 conjugate as the liver is not known to express EGFR. To obtain the maximal therapeutic effect, fluorescence caused by binding of the APC should be high in tumor and low in background. Fluorescence of TCC SH tumor was high after APC injection, while fluorescence signal of background, including liver, decreased beginning 9 hours after APC injection. Thus, we used 1 day of incubation with APC to get the maximal difference between tumor and background normal tissue.

### Histological analysis

**Figure 4:**
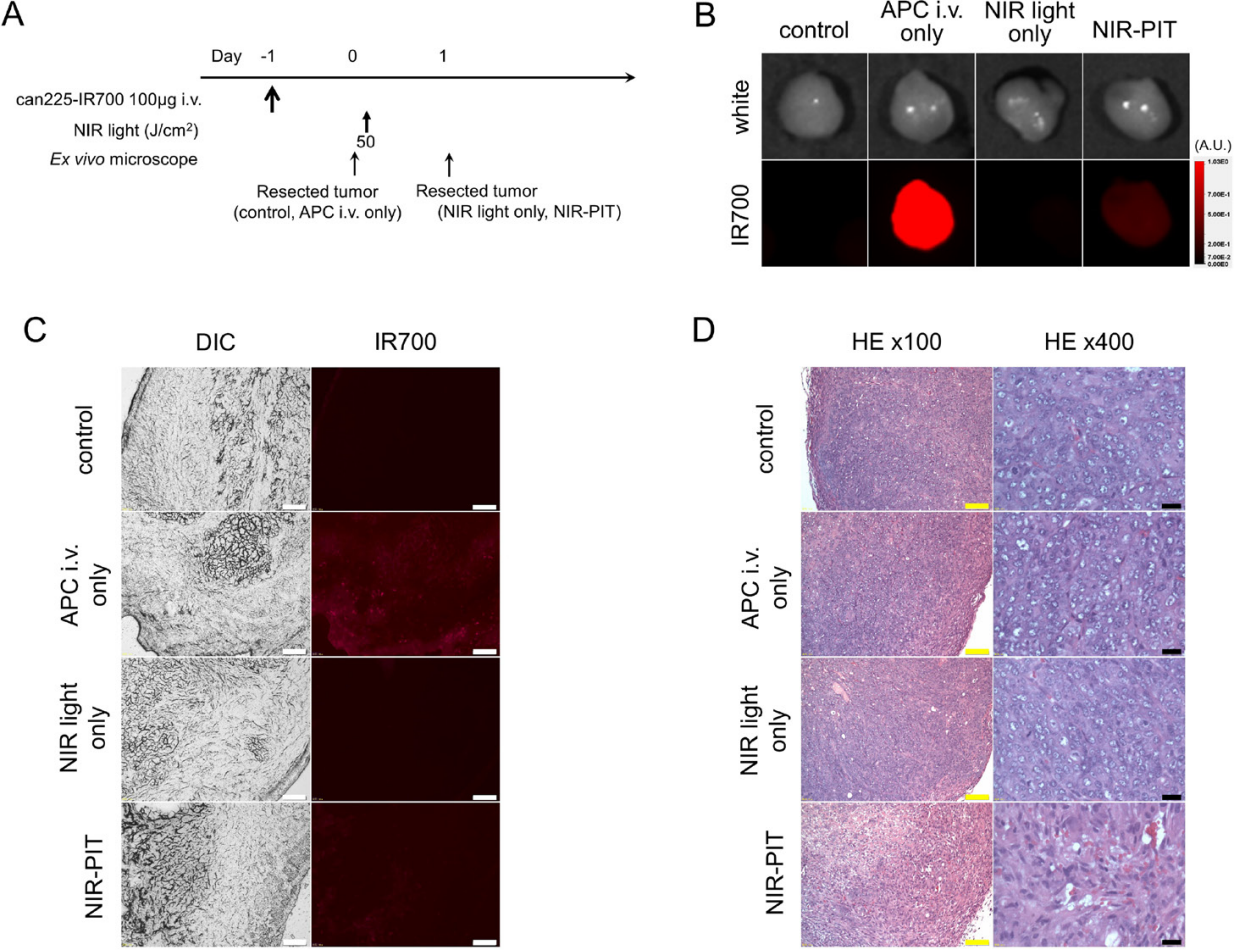
*In vivo* histological fluorescence distribution, and histological NIR-PIT effect (**A**) The regimen of NIR-PIT. (**B**) Fluorescence images of resected TCC SH tumors. White light images (upper) and IR700 fluorescence image (lower). High fluorescence intensity was shown in TCC SH tumor 24 h after injection of can225-IR700, but the fluorescence decreased 24 h after NIR-PIT. No IR700 fluorescence signal was observed in control tumor and NIR light only tumor. (**C**) DIC and fluorescence microscopy images of TCC SH tumor xenografts. High fluorescence intensity was shown in TCC SH tumor 24 h after injection of can225-IR700, but the fluorescence decreased 24 h after NIR-PIT. There were no IR700 fluorescence signals in the tumors without can225-IR700 injection (control tumor and NIR light only tumor). White scale bars = 100 µm. (**D**) Resected tumor stained with hematoxylin and eosin (H&E). A few scattered clusters of damaged tumor cells were seen within a background of diffuse cellular necrosis and micro-hemorrhage after NIR-PIT, while no obvious damage was observed in the control groups, including those receiving can225-IR700 i.v. only (APC i.v. only) or in mice receiving NIR light exposure only (NIR light only). Yellow scale bars = 100 µm. Black scale bars = 20 µm.

The treatment and imaging regimen is shown in Figure 4A. High fluorescence intensity was shown in resected tumors 24 h post can225-IR700 injection, compared with that in control and NIR light only tumors. The majority of fluorescence signal in tumors disappeared 24 h after NIR-PIT in resected tumors (Figure 4B). In frozen histologic specimens, high fluorescence intensity was also shown in tumors 24 h after can225-IR700 injection, and the signal decreased 24 h after NIR-PIT (Figure 4C). There were no fluorescence signals in the tumors without can225-IR700 injection (control tumor and NIR light only tumor) (Figure 4C). Hematoxylin and eosin (H&E) staining of NIR-PIT treated TCC SH tumors revealed diffuse necrosis and micro-hemorrhage, with scattered clusters of live but damaged tumor cells, while no obvious damage was observed in the control tumors, tumors receiving only can225-IR700 but no light, and the tumors exposed to NIR light only, without can225-IR700 injection (Figure 4D).

### *In vivo* NIR-PIT

**Figure 5:**
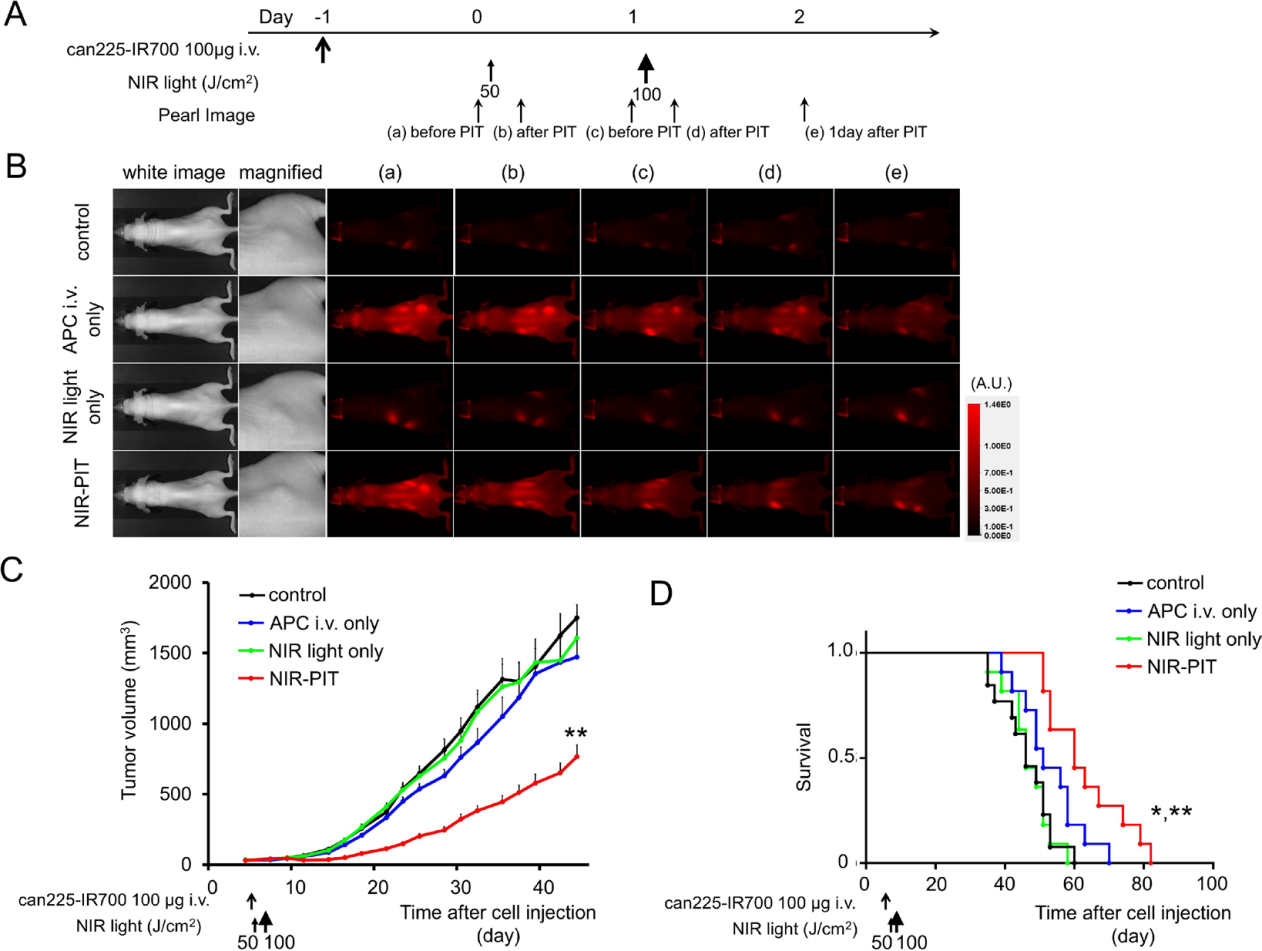
*In vivo* effect of NIR-PIT for TCC SH tumor (**A**) NIR-PIT regimen. Fluorescence images were obtained at each time point as indicated. (**B**) *In vivo* fluorescence real-time imaging of tumor-bearing mice in response to NIR-PIT. One day after injection of can225-IR700, the tumors showed higher fluorescence intensity than did the tumor without can225-IR700 (control and NIR light only group). The tumor treated by NIR-PIT showed decreasing IR700 fluorescence immediately after NIR-PIT. On the other hand, the IR700 fluorescence signal gradually decreased over the following days in tumors receiving can225-IR700 i.v. but not NIR light exposure (APC i.v. only). (**C**) Tumor growth was significantly inhibited in the NIR-PIT treatment group with can225-IR700 (*n* ≧ 10, ^**^p < 0.001 vs other three groups, Tukey’s test with ANOVA). (**D**) Significantly prolonged survival was observed in the NIR-PIT treatment group (*n* ≧ 10, ^**^p < 0.001 vs other three groups, by Log-rank test).

The treatment and imaging regimen is shown in Figure 5A. One day after injection of can225-IR700, the tumors showed higher fluorescence intensity than did the tumors with no can225-IR700 (Figure 5B). After exposure to 50 J/cm^2^ of NIR light, IR700 fluorescence signal of the tumors decreased due to dying cells and partial photo-bleaching, while the IR700 fluorescence gradually decreased over the following days in tumors receiving can225-IR700 without NIR light exposure (Figure 5B). Tumor growth was significantly inhibited in the NIR-PIT treatment group compared with the three control groups (*p* < 0.001) (Figure 5C), and significantly prolonged survival was achieved in the NIR-PIT group (*p* < 0.001 vs other three control groups) (Figure 5D). No significant therapeutic effect was observed in the control groups, including those receiving can225-IR700 i.v. only or in mice exposed to NIR light only. There was no skin necrosis or toxicity attributable to can225-IR700 in any group.

## DISCUSSION

EGFR protein overexpression in TCC/bladder cancer has been linked to TCC grade, stage, and survival outcomes [25–29]. Thus, EGFR is an important target for antibody therapies of invasive bladder cancer in humans [30]. However, randomized clinical trial using dual inhibitors of EGFR and human epidermal growth factor receptor 2 (HER2), lapatinib, failed to improve outcomes in invasive bladder cancer patients [31]. Moreover, it was reported that the addition of lapatinib to chemotherapy as part of neoadjuvant therapy for invasive bladder cancer was limited by excessive treatment-related toxicity [32]. Therefore, to improve the outcome of invasive bladder cancer, treatments with fewer side effects are urgently needed.

NIR-PIT has shown excellent selectivity for antigen expressing tumor cells with minimal off-target effects. As shown in this study, the conjugate can225-IR700 proved to be an effective agent for treating an EGFR-expressing canine invasive bladder cancer cell line with NIR-PIT. NIR-PIT with can225-IR700 led to rapid cell death *in vitro* and tumor growth reduction and survival improvement *in vivo*. Thus, a caninized version of cetuximab-IR700, can225IgG [22], could be an effective platform for NIR-PIT in EGFR expressing canine invasive bladder cancer. Compared with other therapies, antibody-based phototherapy has several advantages over conventional approaches. For instance, it is minimally invasive and can be applied repeatedly [33]. It appears to induce a strong immune response that appears to be limited to the treated tumor volume, and minimal side effects have been seen in early-phase clinical trials. Thus, the ability of can225IgG-IR700 to act as a NIR-PIT agent opens up the possibility of testing in immune-competent tumor-bearing dogs.

It is important to differentiate NIR-PIT from photodynamic therapy (PDT) that has been used for several decades. The photosensitizers used in PDT are very hydrophobic and therefore, difficult to target. For instance, when antibodies were conjugated to PDT photosensitizers, these conjugates were immediately recognized by the reticuloendothelial system and accumulated in the liver, resulting in minimal accumulation in target tumors when applied intravenously. In contrast, NIR-PIT employs a water-soluble silica-phthalocyanine dye, which is conjugated to an antibody to form an APC with a biodistribution similar to non-conjugated parental antibodies in the body. Therefore, once injected intravenously, the APC gradually distributes into tumors within days, resulting in sufficient accumulation within cancer cells to kill the cells after NIR light exposure. Our previous study has shown NIR-PIT to be highly cell-specific, therefore, non-target cells immediately adjacent to targeted cells show minimal toxic effect [1].

Based on fluorescence TBR, the can225-IR700 conjugate achieved sufficient accumulation for NIR-PIT (Figure 3). Our results confirm that can225IgG bound to canine EGFR on cells specifically and was internalized within 6 hours of incubation in EGFR expressing cancer cells (Figure 2). Of course, for the treatment of bladder cancer, for instance, it is also necessary to modify existing cystoscopic equipment to deliver light to the bladder. The dog model patient also permits a more realistic appraisal of both efficacy and side effects than is possible in mouse model, wherein standardized metrics, adopted from human clinical trials, are applied for assessment of tumor response and attribution and grading of systemic adverse events [34, 35].

An important aspect of NIR-PIT is its immunogenic nature. Cells treated with NIR-PIT undergo rapid volume expansion leading to rupture of the cell membrane and extrusion of cell contents into the extracellular space [17]. This causes an immunogenic cell death rather than apoptotic cell death which is induced by most other cancer therapies [36, 37]. After NIR-PIT dying cells release neoantigens and signaling moieties including calreticulin, ATP, and HMGB1 that effectively promote maturation of immature dendritic cells [17]. These matured dendritic cells are capable not only of digesting the cancer cell debris but also present cancer-specific antigens to T-effector cells [17]. It is anticipated that immunogenic cell death will augment the therapeutic effect of NIR-PIT as it selectively kills target cells while non-target cells immediately adjacent, including effector immune cells, show no toxic effects [1]. Therefore, NIR-PIT could effectively enforce host tumor immunity. Pet dogs trials of NIR-PIT for naturally occurring invasive bladder cancer are appropriate to clarify the immunological response after NIR-PIT prior to human clinical trial of bladder cancer.

This study has several limitations. We performed one injection of the can225-IR700 with two exposures of NIR light in order to perform a proof-of-principle study for demonstrating that NIR-PIT with can225-IR700 was effective to treat EGFR-expressing canine cancer models. Clearly, repeated dosing of the APC with repeated NIR light exposure could have improved the therapeutic effect [21, 38]. It would be desirable to extend these studies to multiple doses of the APC and NIR light. This study was also done in xenograft model of nude mice. In mice, NIR light could be delivered into deep tumor, such as bladder cancer, using surface illumination, while this is impossible in dogs and humans. To deliver NIR light, direct exposure of NIR light would be necessary using either direct illumination during surgery or using fibro-optic diffusers via cystoscopes or inserted needles. Finally, the current xenograft model does not sufficiently represent clinical cancers. Surgically-implanted orthotopic tumor models are superior to xenografted tumor models [39–42], yet consistently producing orthotopic implants with mouse surgery requires highly trained skills. Therefore, in this proof-of-principle study of NIR-PIT targeting canine bladder cancer, we chose this simple subcutaneous xenograft tumor model.

## CONCLUSIONS

IR700 conjugate with the caninized version of cetuximab, can225IgG, accumulated in high EGFR-expressing canine invasive transitional bladder cancer cells. Subsequent NIR-PIT using can225-IR700 induced remarkable therapeutic responses after only a single injection of the conjugate and two NIR light exposures in an EGFR-expressing canine xenograft tumor model. Thus, NIR-PIT utilizing EGFR as the targeting antigen for the can225-IR700 shows promise as a new treatment modality for naturally-occurring canine invasive TCC in pet dogs as a prelude to translation into humans.

## MATERIALS AND METHODS

### Reagents

Water soluble, silica-phthalocyanine derivative, IRDye 700DX NHS ester was obtained from LI-COR Biosciences (Lincoln, NE, USA). A caninized version of human anti-EGFR antibody (cetuximab), can225IgG, was engineered and produced as previously described [22]. All other chemicals were of reagent grade.

### Can225IgG expression and purification

Can225IgG was expressed as previously described [22]. In short, vectors containing the EGFR-specific γ-C heavy and κ-light chain sequences were co-transfected into CHO-DUKX-B11 cells and expressed under serum-free conditions in ProCHO 5 medium (Lonza, Basel, Switzerland) supplemented with 4 mM L-glutamine. Subsequently, can225IgG was affinity-purified from cell culture supernatant with a HiTrap Protein G HP column (GE Healthcare, Piscataway, NJ, USA) using an ÄKTA explorer FPLC system (GE Healthcare, Piscataway, NJ, USA).

### Synthesis of IR700-conjugated can225

Conjugation of dyes with mAb was performed according to a previous report [1]. In brief, can225IgG (1.0 mg, 6.8 nmol) was incubated with IR700 NHS ester (66.0 μg, 33.8 nmol) in 0.1 M Na_2_HPO_4_ (pH 8.6) at room temperature for 1 h. The mixture was purified with a Sephadex G25 column (PD-10; GE Healthcare, Piscataway, NJ, USA). The protein concentration was determined with Coomassie Plus protein assay kit (Thermo Fisher Scientific Inc, Rockford, IL, USA) by measuring the absorption at 595 nm with UV-Vis (8453 Value System; Agilent Technologies, Santa Clara, CA, USA). The concentration of IR700 was measured by absorption at 689 nm to confirm the number of fluorophore molecules per mAb. The synthesis was controlled so that an average of two IR700 molecules was bound to a single antibody. We abbreviate IR700 conjugated to can225IgG as can225-IR700. As a quality control for the conjugate, we performed SDS-PAGE. Conjugate was separated by SDS-PAGE with a 4–20% gradient polyacrylamide gel (Life Technologies, Gaithersburg, MD, USA). A standard marker (Crystalgen Inc., Commack, NY, USA) was used as a protein molecular weight marker. After electrophoresis at 80 V for 2.5 h, the gel was imaged with a Pearl Imager (LI-COR Biosciences, Lincoln, Nebraska, USA) using a 700 nm fluorescence channel. We used diluted can225IgG as a control. The gel was stained with Colloidal Blue staining to determine the molecular weight of conjugate.

### Cell culture

Three canine transitional bladder cancer cell lines, K9TCC original (minimal EGFR expression), K9TCC-PU AxA (low EGFR expression) and K9TCC-PU Sh (intermediate EGFR expression) were kindly provided by Dr. Deborah W. Knapp (Purdue University Center for Cancer Research, Purdue University, West Lafayette, IN, US). All cell lines were established from tumor samples of dogs with invasive transitional cell carcinoma (TCC). We abbreviate these cell lines as TCC original, TCC AXA, and TCC SH. Cells were grown in DMEM/F12 (Life Technologies, Gaithersburg, MD, USA) supplemented with 10% fetal bovine serum (Life Technologies) in tissue culture flasks in a humidified incubator at 37° C in an atmosphere of 95% air and 5% carbon dioxide.

### Flow cytometry

To verify *in vitro* can225-IR700 binding, fluorescence from cells after incubation with can225-IR700 was measured using a flow cytometer (FACS Calibur, BD BioSciences, San Jose, CA, USA) and CellQuest software (BD BioSciences). TCC original, TCC AXA and TCC SH cells (2 × 10^5^) were seeded into 12 well plates and incubated for 24 h. Cell media was replaced with fresh culture media containing 3 µg/mL of can225-IR700 and incubated for 6 h at 37° C. After washing with phosphate buffered saline (PBS), PBS was added. A 488-nm argon ion laser was used for excitation. Signals from cells were collected with a 653-669 nm band-pass filter. To validate the specific binding of the conjugated antibody, excess naked antibody (30 µg) was used to block can225-IR700 uptake.

### Fluorescence microscopy

To detect the antigen specific localization and effect of NIR-PIT, fluorescence microscopy was performed (BX61; Olympus America, Inc., Melville, NY, USA). Ten thousand TCC original, TCC AXA and TCC SH cells were seeded on cover-glass-bottomed dishes and incubated for 24 h. Can225-IR700 was then added to the culture medium at 3 µg/mL and incubated for 6 h at 37° C. After incubation, the cells were washed with PBS. The filter set to detect IR700 consisted of a 590–650 nm excitation filter, a 665–740 nm band pass emission filter. Transmitted light differential interference contrast (DIC) images were also acquired.

### *In vitro* NIR-PIT

The cytotoxic effects of NIR-PIT with can225-IR700 were determined by flow cytometric propidium iodide (PI) (Life Technologies) staining, which can detect compromised cell membranes. Two hundred thousand TCC original, TCC AXA and TCC SH cells were seeded into 12 well plates and incubated for 24 h. Medium was replaced with fresh culture medium containing 3 µg/ml of can225-IR700 and incubated for 6 h at 37° C. After washing with PBS, PBS was added, and cells were irradiated with a red light-emitting diode (LED), which emits light at 690 ± 20 nm wavelength (L690-66-60; Marubeni America Co., Santa Clara, CA, USA) at a power density of 50 mW/cm^2^ as measured with an optical power meter (PM 100, Thorlabs, Newton, NJ, USA). Cells were scratched 1 h after treatment. PI was then added to the cell suspension (final 2 µg/mL) and incubated at room temperature for 30 min, followed by flow cytometry. A 488-nm argon ion laser was used for excitation. Signals from cells were collected with a 650 nm long-pass filter for PI.

### Animal and tumor models

All *in vivo* procedures were conducted in compliance with the Guide for the Care and Use of Laboratory Animal Resources (1996), US National Research Council, and approved by the local Animal Care and Use Committee. Six to eight week old female homozygote athymic nude mice were purchased from Charles River (NCI-Frederick, Frederick, MD, USA). During the procedure, mice were anesthetized with inhaled 3–5% isoflurane and/or via intraperitoneal injection of 1 mg of sodium pentobarbital (Nembutal Sodium Solution, Ovation Pharmaceuticals Inc., Deerfield, IL, USA). In order to determine tumor volume, the greatest longitudinal diameter (length) and the greatest transverse diameter (width) were measured with an external caliper. Tumor volumes were based on caliper measurements and were calculated using the following formula; tumor volume = length × width^2^ × 0.5. Body weight was also measured. Mice were monitored daily for their general health. The presence of skin necrosis or toxicity attributable to the can225-IR700 was evaluated carefully noting skin color, general health, weight loss and loss of appetite. Tumor volumes were measured three times a week until the tumor volume reached 2000 mm^3^, whereupon the mice were euthanized with inhalation of carbon dioxide gas.

### *In vivo* fluorescence imaging studies

TCC SH cells (2 × 10^6^) were injected subcutaneously in the right dorsum of the mice. Tumors were studied after they reached volumes of approximately 50 mm^3^. Serial ventral and dorsal fluorescence images of IR700 were obtained with a Pearl Imager using a 700 nm fluorescence channel before and 0, ½, 1, 2, 3, 4, 5, 6, 9, 12, 24, 48, 72, 96, 120, 144, and 168 hours after i.v. injection of 100 µg of can225-IR700 via the tail vein. Pearl Cam Software (LICOR Biosciences, Lincoln, NE, USA) was used for analyzing fluorescence intensities. Regions of interests (ROIs) were placed on the tumor and liver to quantify the fluorescence intensities. ROIs were also placed in the adjacent non-tumor region as background (left dorsum and lower abdomen). Average fluorescence intensity of each ROI was calculated. Target-to-background ratios (TBR = fluorescence intensities of target / fluorescence intensities of background) were also calculated (*n* = 10).

### Histological analysis

To detect the antigen-specific micro-distribution in the tumor, fluorescence microscopy was performed. Tumor xenografts were excised from mice without treatment (control), 24 h after injection of can225-IR700 (APC i.v. only), 24 h after NIR exposure without can225-IR700 (NIR light only) and 24 h after NIR-PIT (NIR-PIT). Fluorescence images, as well as white light images, of extracted tumors were obtained using a Pearl Imager with a 700 nm fluorescence channel. Then, extracted tumors were frozen with optimal cutting temperature (OCT) compound (SAKURA Finetek Japan Co., Tokyo, Japan) and frozen sections (10 µm thick) were prepared. Fluorescence microscopy was performed using the BX61 microscope with the following filters: excitation wavelength 590 to 650 nm, emission wavelength 665 to 740 nm long pass for IR700 fluorescence. DIC images were also acquired. To evaluate histological changes, bright-field microscopy was also performed. Extracted tumors were fixed with 10% formalin and serial 10 µm slice sections were fixed on a glass slide with H&E staining.

### *In vivo* NIR-PIT

TCC SH cells (2 × 10^6^) were injected subcutaneously in the right dorsum of each mouse. Tumors were studied after they reached volumes of approximately 50 mm^3^. To examine the therapeutic effect of *in vivo* NIR-PIT on TCC SH tumors, tumor-bearing mice were randomized into 4 groups of at least 10 animals per group for the following treatments: (1) no treatment (control); (2) 100 µg of can225-IR700 i.v. only, no NIR light exposure (APC i.v. only); (3) NIR light exposure only, NIR light exposure was administered at 50 J/cm^2^ on day 1 and 100 J/cm^2^ on day 2 without can225-IR700 i.v. (NIR light only); (4) 100 µg of can225-IR700 i.v., NIR light exposure was administered at 50 J/cm^2^ on day 1 and 100 J/cm^2^ on day 2 after injection (NIR-PIT). Serial fluorescence images, as well as white light images, were obtained using a Pearl Imager with a 700 nm fluorescence channel.

### Statistical analysis

Data are expressed as means ± standard error of mean (SEM) from a minimum of five experiments, unless otherwise indicated. Statistical analyses were carried out using GraphPad Prism version 7 (GraphPad Software, La Jolla, CA, USA). For multiple comparisons, a one-way analysis of variance (ANOVA) followed by the Tukey’s correction for multiple comparisons was used. The cumulative probability of survival based on volume (2000 mm^3^) were estimated in each group with a Kaplan–Meier survival curve analysis, and the results were compared with use of the log-rank test. Student’s *t* test was used to compare the treatment effects with that of control. A *p*-value of < 0.05 was considered statistically significant.

The research was also supported by the National Institute for Health Research (NIHR) Biomedical Research Centre (BRC) based at Guy’s and St Thomas’ NHS Foundation Trust and King’s College London (IS-BRC-1215-20006). The views expressed are those of the author(s) and not necessarily those of the NHS, the NIHR or the Department of Health. The authors acknowledge support by Breast Cancer Now (147), working in partnership with Walk the Walk.

JFS was supported by the Austrian Science Fund FWF, grant W1205-B09 (CCHD) to EJJ.

## Abbreviations

ANOVA: one-way analysis of variance
APC: antibody-photo-absorber conjugate
DIC: differential interference contrast
EGFR: epidermal growth factor receptor
FDA: Food and Drug Administration
H&E: hematoxylin and eosin
HER2: human epidermal growth factor receptor 2
IR700: IRDye700DX
LED: light-emitting diode
mAb: monoclonal antibodies
NIR: near-infrared
OCT: optimal cutting temperature
PBS: phosphate buffered saline
PDT: photodynamic therapy
PI: propidium iodide
PIT: photoimmunotherapy
ROI: regions of interest
SEM: standard error of mean
SDS-PAGE: sodium dodecyl sulfate-polyacrylamide gel electrophoresis
TBR: target-to-background ratio
TCC: transitional cell carcinoma
US: United States
